# Sex differences in hypercholesterolemia management (2002−2022): evidence from the Swiss National Health Surveys

**DOI:** 10.1016/j.pmedr.2025.103266

**Published:** 2025-10-08

**Authors:** Shun Yi, Roxane de La Harpe, Pedro Marques-Vidal

**Affiliations:** a Department of Medicine, Internal Medicine, Lausanne University Hospital (CHUV) and University of Lausanne, Lausanne, Switzerland; b Faculty of Biology and Medicine, University of Lausanne, Lausanne, Switzerland

**Keywords:** Sex, Cardiovascular disease, Epidemiology, Hypercholesterolemia, National survey

## Abstract

**Objective:**

To investigate sex differences in hypercholesterolemia management (screening, diagnosis, treatment, control) in Switzerland (2002–2022); secondarily, to examine whether these disparities changed over time and varied by age.

**Methods:**

Data from five nationally representative Swiss Health Surveys were used (*n* = 72,804; 53.9 % female). Multivariable logistic regressions were adjusted for demographic, socioeconomic, and lifestyle covariates. Age-stratified analyses used >50 years as a proxy for menopause.

**Results:**

After multivariable adjustment, no significant sex differences in screening were observed except in 2022. Across all survey years, females were consistently less likely to be diagnosed with hypercholesterolemia (e.g., OR 0.66, 95 % CI 0.61, 0.70 in 2022) or to be treated once diagnosed (e.g., OR 0.77, 95 % CI 0.68, 0.88 in 2022), with no evidence of narrowing over time. Among treated participants, cholesterol control was initially higher in females (2002–2007) but showed no sex difference by 2022. Age-stratified analyses indicated that the magnitude and direction of sex disparities varied by age.

**Conclusion:**

This study indicates age-specific sex differences across all stages of self-reported hypercholesterolemia management in Switzerland over two decades.

## Highlights

• Analyzed 20 years of national survey data on hypercholesterolemia management.
• Identified sex gaps in screening, diagnosis, treatment, and control.
• Used age > 50 as a menopause proxy to examine age-specific disparities.
• Results showed undertreatment of older females despite universal healthcare.

## 1 Introduction

Cardiovascular disease (CVD) remains the leading cause of mortality globally (Lindstrom et al., 2022). Despite being a heavy burden in the healthcare system, a substantial proportion of CVD is preventable through effective management of modifiable risk factors, notably hypercholesterolemia. Elevated low-density lipoprotein cholesterol (LDL-C) is a primary clinical marker of hypercholesterolemia, and evidence shows that each 1.0 mmol/L reduction in LDL-C is associated with a 15 % decrease in CVD mortality (Khan and Michos, 2020).

Despite universal healthcare coverage, the management of hypercholesterolemia remains suboptimal, even in high-income countries like Switzerland (Chekanova et al., 2023). Accumulated evidence indicates that missed opportunities for effective hypercholesterolemia management are associated with increased risk of CVD (Ryou et al., 2021). Sex disparities further complicate this landscape, with females or males experiencing distinct challenges, particularly due to social factors related to healthcare inequities, such as differences in health literacy and socioeconomic status (Kesic et al., 2022; Lopez-Ferreruela et al., 2025). These factors may influence risk perception and access to care; as a result, lower screening rates and reduced statin initiation among females were observed (Rachamin et al., 2021; Marques-Vidal et al., 2023; Rochat et al., 2023). While existing studies highlight sex differences at specific stages, little is known about how these disparities have evolved over time at the population level, particularly across stages of care.

With its long-standing national health survey program, Switzerland offers a unique opportunity to examine these trends over time in a population-based setting. This study primarily aimed to investigate sex differences in the screening, diagnosis, treatment, and control of hypercholesterolemia in Switzerland. Secondarily, we assessed whether these disparities changed over time and whether they varied by age.

## 2 Methods

### 2.1 Database and sampling

This study utilized data from five consecutive Swiss Health Surveys (SHS) conducted in 2002, 2007, 2012, 2017, and 2022. The SHS is a nationally representative, cross-sectional survey implemented every five years by the Swiss Federal Statistical Office. Participants were selected through multi-stratified random sampling from private Swiss households. All participants were aged ≥15 years. Detailed information on the survey methodology and the complete questionnaire is provided by the Swiss Federal Statistical Office (Swiss Federal Statistical Office, 2023; Swiss Federal Statistical Office, 2024).

Of 104,126 participants in the 2002–2022 SHSs, we excluded those with missing outcome information on cholesterol screening (*n* = 30,244; 29.1 %) and those with missing data on one or more covariates (*n* = 1078; 1.0 %), yielding an analytic sample of 72,804 participants (69.9 %; 53.9 % female; **Supplementary Fig. 1**). Compared with included participants, excluded individuals were more often younger, professionally active, single, non-Swiss, less educated, and of normal weight (**Supplementary Table 1**).

Ethical approval was not required, as the SHS is mandated by the Swiss Federal Government, and all data were anonymized before analysis.

### 2.2 Exposure

Sex was self-reported as male or female.

### 2.3 Outcomes

Self-reported data on cholesterol screening, diagnosis, treatment, and control were extracted from the structured questionnaire (**Supplementary Table 2**). Screening was defined as having ever had a cholesterol measurement (previous screening) or having had one within the last 12 months (screening last 12 months). Screening and diagnosis were calculated for all respondents. Treatment was defined as self-reported use of cholesterol medication in the last 7 days (yes/no) among those with a diagnosis. Control was assessed among treated participants, coded as “controlled” if cholesterol was reported normal and “uncontrolled” if reported too high.

### 2.4 Covariates

We considered the following variables as potential confounders, all of which were self-reported in the SHS questionnaires. Age at the time of the survey was categorized into the following groups: 15–24, 25–44, 45–64, and ≥ 65 years. Nationality was classified as Swiss or Other. Education was grouped as primary, secondary, or tertiary. Workforce status was categorized as inactive, jobless, or active. Civil status was categorized as single, married, divorced, or widowed. Smoking status was categorized as never, former, or current smoker. Alcohol consumption was categorized into none, low (≤20 g/day in males, ≤10 g/day in females), moderate (20–40 g/day in males, 10–20 g/day in females), average (40–60 g/day in males, 20–40 g/day in females), and high (>60 g/day in males, >40 g/day in females). Vigorous physical activity was defined as physical activity that causes sweating (e.g., jogging) and was categorized as none, 1–2 days/week, or ≥ 3 days/week. Body Mass Index (BMI) was calculated from self-reported height and weight and classified according to WHO standards: underweight (<18.5 kg/m^2^), normal weight (18.5–24.9 kg/m^2^), overweight (25–29.9 kg/m^2^), and obese (≥30 kg/m^2^). The region was coded according to the different administrative regions in Switzerland.

### 2.5 Statistical analysis

For each survey year, we performed descriptive analyses of participant characteristics and outcomes to assess sex differences, using Pearson's chi-square test.

To assess sex differences in hypercholesterolemia management, we first estimated a pooled multivariable logistic regression model including sex as the main exposure, adjusting for survey year and all covariates, to obtain the overall female-to-male adjusted ORs. To evaluate whether sex disparities varied over calendar time, we then included a sex×year (continuous) interaction term in the pooled models and assessed its significance using likelihood-ratio tests (p-interaction). Finally, for descriptive purposes, we also ran separate logistic regression models for each survey year to obtain year-specific adjusted ORs and 95 % CIs. To assess whether sex differences varied by age, we first tested sex×age interaction terms in pooled logistic regression models and reported the corresponding p-interaction values, obtained using likelihood-ratio tests. When evidence of effect modification was observed, we conducted stratified analyses by age group (≤50 vs >50 years), using age > 50 as a proxy for the post-menopausal period in females based on epidemiological evidence (Heer et al., 2020).

Given that a substantial portion of the sample was excluded, a sensitivity analysis was performed using inverse probability weighting (IPW) (Mansournia and Nazemipour, 2024). In this approach, the probability of inclusion in the analysis was modelled based on variables that significantly differed between included and excluded participants. The inverse of this probability was then applied to multivariable analysis to account for selection bias due to missing data.

Statistical analysis was conducted using Stata 18 (Stata Corp., College Station, TX, USA). Statistical significance was considered for a two-sided test with *p* < 0.05.

## 3 Results

### 3.1 Sample selection and characteristics

The demographic and lifestyle characteristics of the selected cases were summarized in Table 1. Within each survey year, females were more likely than males to be Swiss nationals, have lower educational attainment, be professionally inactive, and be divorced or widowed. Females also reported lower prevalences of smoking, alcohol consumption, and overweight/obesity, whereas males more frequently reported vigorous physical activity.

**Table 1:** Demographic and lifestyle characteristics by sex among participants aged ≥15 years in Switzerland, 2002–2022.

	**2002**		**2007**		**2012**		**2017**		**2022**	
**Characteristic**	**Male** **N (%)**	**Female** **N (%)**	**Male** **N (%)**	**Female** **N (%)**	**Male** **N (%)**	**Female** **N (%)**	**Male** **N (%)**	**Female** **N (%)**	**Male** **N (%)**	**Female** **N (%)**
**Sample size**	5285	6695	5126	6501	7330	8092	8033	8797	7826	9119

**Age group**										
15–24	340 (6.4)	348 (5.2)	407 (7.9)	440 (6.8)	925 (12.6)	920 (11.4)	**998 (12.4)**	**1054 (12.0)**	**714 (9.1)**	**767 (8.4)**
25–44	1768 (33.5)	2143 (32.0)	1508 (29.4)	1912 (29.4)	1862 (25.4)	2174 (26.9)	**1991 (24.8)**	**2324 (26.4)**	**1711 (21.9)**	**2095 (23.0)**
45–64	1939 (36.7)	2441 (36.5)	1905 (37.2)	2223 (34.2)	2734 (37.3)	2981 (36.8)	**3025 (37.7)**	**3241 (36.8)**	**2987 (38.2)**	**3396 (37.2)**
65+	1238 (23.4)	1763 (26.3)	1306 (25.5)	1926 (29.6)	1809 (24.7)	2017 (24.9)	**2019 (25.1)**	**2178 (24.8)**	**2414 (30.8)**	**2861 (31.4)**

**Nationality**										
Swiss	4612 (87.3)	5955 (89.0)	4429 (86.4)	5812 (89.4)	6031 (82.3)	6891 (85.2)	6179 (76.9)	7156 (81.4)	6270 (80.1)	7476 (82.0)
Other	673 (12.7)	740 (11.0)	697 (13.6)	688 (10.6)	1299 (17.7)	1201 (14.8)	1854 (23.1)	1641 (18.6)	1556 (19.9)	1643 (18.0)

**Education level**										
Primary	715 (13.5)	1735 (25.9)	687 (13.4)	1728 (26.6)	1134 (15.5)	1820 (22.5)	1303 (16.2)	1900 (21.6)	856 (10.9)	1466 (16.1)
Secondary	3176 (60.1)	4311 (64.4)	2726 (53.2)	3762 (57.9)	3555 (48.5)	4542 (56.1)	3706 (46.1)	4600 (52.3)	3404 (43.5)	4504 (49.4)
Tertiary	1394 (26.4)	649 (9.7)	1713 (33.4)	1011 (15.5)	2641 (36.0)	1730 (21.4)	3024 (37.7)	2297 (26.1)	3566 (45.6)	3149 (34.5)

**Workforce status**										
Inactive	1432 (27.1)	2899 (43.3)	1461 (28.5)	2708 (41.7)	2081 (28.4)	3096 (38.2)	2247 (28.0)	3277 (37.3)	2511 (32.1)	3655 (40.1)
Jobless	88 (1.7)	132 (2.0)	113 (2.2)	146 (2.2)	116 (1.6)	152 (1.9)	174 (2.2)	200 (2.3)	126 (1.6)	137 (1.5)
Active	3763 (71.2)	3664 (54.7)	3551 (69.3)	3646 (56.1)	5133 (70.0)	4843 (59.9)	5608 (69.8)	5317 (60.5)	5185 (66.3)	5324 (58.4)

**Civil status**										
Single	1299 (24.6)	1428 (21.4)	1288 (25.1)	1445 (22.3)	2107 (28.7)	2042 (25.2)	2288 (28.5)	2358 (26.8)	2232 (28.5)	2316 (25.4)
Married	3246 (61.4)	3462 (51.7)	2988 (58.3)	3120 (48.0)	4438 (60.6)	4309 (53.3)	4882 (60.8)	4768 (54.2)	4661 (59.6)	4963 (54.4)
Divorced	497 (9.4)	837 (12.5)	576 (11.3)	860 (13.2)	570 (7.8)	978 (12.1)	684 (8.5)	964 (11.0)	712 (9.1)	1097 (12.0)
Widowed	242 (4.6)	966 (14.4)	273 (5.3)	1073 (16.5)	213 (2.9)	759 (9.4)	179 (2.2)	707 (8.0)	221 (2.8)	743 (8.2)

**Smoking status**										
Never	1996 (37.8)	3720 (55.5)	2077 (40.5)	3681 (56.6)	3101 (42.3)	4543 (56.1)	3556 (44.3)	5041 (57.3)	3715 (47.4)	5301 (58.1)
Former	1547 (29.3)	1203 (18.0)	1516 (29.6)	1289 (19.8)	2048 (27.9)	1592 (19.7)	2200 (27.4)	1738 (19.8)	2187 (28.0)	2054 (22.5)
Current	1742 (32.9)	1772 (26.5)	1533 (29.9)	1531 (23.6)	2181 (29.8)	1957 (24.2)	2277 (28.3)	2018 (22.9)	1924 (24.6)	1764 (19.4)

**Alcohol consumption**										
No consumption	668 (12.6)	2045 (30.5)	486 (9.5)	1408 (21.7)	736 (10.0)	1655 (20.5)	969 (12.1)	1906 (21.7)	834 (10.7)	1713 (18.8)
Low risk	3248 (61.5)	3331 (49.8)	3472 (67.7)	3804 (58.5)	5104 (69.6)	4922 (60.8)	5519 (68.7)	5310 (60.3)	5674 (72.5)	5947 (65.2)
Moderate risk	912 (17.3)	985 (14.7)	847 (16.5)	959 (14.8)	1077 (14.7)	1142 (14.1)	1111 (13.8)	1187 (13.5)	989 (12.6)	1149 (12.6)
Average risk	261 (4.9)	247 (3.7)	185 (3.6)	269 (4.1)	238 (3.3)	316 (3.9)	261 (3.3)	332 (3.8)	204 (2.6)	255 (2.8)
High risk	196 (3.7)	87 (1.3)	136 (2.7)	61 (0.9)	175 (2.4)	57 (0.7)	173 (2.1)	62 (0.7)	125 (1.6)	55 (0.6)

**Vigorous physical activity**										
None	1822 (34.5)	2960 (44.2)	1600 (31.2)	2605 (40.1)	2258 (30.8)	3295 (40.7)	2365 (29.4)	3124 (35.5)	2613 (33.4)	3803 (41.7)
1–2 days/week	1894 (35.8)	2177 (32.5)	1848 (36.1)	2104 (32.4)	2720 (37.1)	2898 (35.8)	2959 (36.9)	3299 (37.5)	2581 (33.0)	2938 (32.2)
≥3 days/week	1569 (29.7)	1558 (23.3)	1678 (32.7)	1792 (27.5)	2352 (32.1)	1899 (23.5)	2709 (33.7)	2374 (27.0)	2632 (33.6)	2378 (26.1)

**BMI category**										
Underweight	55 (1.0)	358 (5.4)	38 (0.7)	360 (5.5)	76 (1.1)	477 (5.9)	99 (1.2)	465 (5.3)	95 (1.2)	490 (5.4)
Normal	2561 (48.5)	4104 (61.3)	2447 (47.7)	3973 (61.1)	3321 (45.3)	4876 (60.3)	3555 (44.3)	5311 (60.4)	3410 (43.6)	5410 (59.3)
Overweight	2141 (40.5)	1641 (24.5)	2130 (41.6)	1551 (23.9)	3022 (41.2)	1927 (23.8)	3330 (41.4)	2097 (23.8)	3188 (40.7)	2151 (23.6)
Obese	528 (10.0)	592 (8.8)	511 (10.0)	617 (9.5)	911 (12.4)	812 (10.0)	1049 (13.1)	924 (10.5)	1133 (14.5)	1068 (11.7)

Results are presented as numbers (percentages) for categorical variables. Between-group comparisons were performed within each survey year using chi-square tests. BMI, Body Mass Index. Besides values in bold corresponding to non-significant (p ≥ 0.05) results, all comparisons are significant at p < 0.05.

### 3.2 Screening

Overall, 89.5 % reported ever being screened, and 63.6 % within the past 12 months. When stratified by year, except for 2002 and 2017, males had higher overall screening coverage than females (**Supplementary Table 3**). However, no difference in 12-month screening proportions was observed between sexes over the years (**Supplementary Table 3**).

After multivariable adjustment, females were overall less likely to have been previously screened (overall adjusted OR (aOR) 0.93, 95 % CI: 0.88, 0.98) but more likely to have been screened in the last 12 months (aOR 1.05, 95 % CI: 1.01, 1.09). When testing for sex×year interaction, there was no evidence that sex disparities in screening varied significantly across survey years (p-interaction = 0.76 for previous screening; p-interaction = 0.05 for screening in the last 12 months). When stratified by year, the aOR for previous screening was mostly below 1.0, although not reaching statistical significance until 2022. For screening in the last 12 months, the aOR across all years was slightly above 1.0, with statistical significance reached in 2022 (**Supplementary Table 4**).

### 3.3 Diagnosis

The overall diagnosis of hypercholesterolemia was around 64 %. When stratified by year, the diagnosis was lower for females than for males across all years (**Supplementary Table 3**).

This finding was supported by multivariable analysis conducted on the overall diagnosis of hypercholesterolemia (aOR 0.65, 95 % CI: 0.63, 0.67) as well as diagnosis stratified by year (**Supplementary Table 4**). There was no evidence of significant variation in this disparity across survey years (sex×year interaction, p-interaction = 0.06).

### 3.4 Treatment

Among those diagnosed, the overall medication prescription was 89.3 %. When stratified by year, females consistently had lower medication prescriptions than males (**Supplementary Table 3**).

In the multivariable analysis, females were overall less likely to receive treatment than males (aOR 0.73, 95 % CI: 0.68, 0.78). The sex×year interaction was not statistically significant (p-interaction = 0.41), indicating that the treatment gap did not vary significantly across survey years. When stratified by year, this difference persisted across all study periods (**Supplementary Table 4**).

### 3.5 Control

Among those treated, overall 94.3 % of participants reported controlled cholesterol levels. When stratified by year, except for 2022, females reported higher control levels than males (**Supplementary Table 3**).

In the multivariable analysis, females were overall more likely to report controlled cholesterol levels (aOR 1.14, 95 % CI: 1.04, 1.26), significant in 2002 and 2007 but not thereafter (**Supplementary Table 4**). There was no evidence of variation in this disparity across survey years (sex×year interaction, p-interaction = 0.25).

In pooled models, sex×age interactions were statistically significant for previous screening (p < 0.01), recent screening (p < 0.01), diagnosis (p < 0.01), treatment (p < 0.01), and control (p < 0.01), indicating that sex disparities differed between younger (≤50 years) and older (>50 years) participants. In Table 2**,** for previous screening, the lower likelihood of screening among females was mainly driven by those >50 years, particularly in 2012 and 2022, while no significant difference was observed between younger participants (age ≤ 50). For recent (last 12 months) screening, the higher likelihood of being screened among younger females than younger males from 2012 onwards contrasted with the lower likelihood of being screened in older females than older males in 2012 and 2017. For both age groups, females had a lower likelihood of reporting a diagnosis of hypercholesterolemia than males across all years. Among those diagnosed, older females consistently had a lower likelihood of receiving treatment compared to older males, while the lower likelihood of being treated among younger females than younger males was only observed in 2012 and 2017. Among treated participants, the higher likelihood of control was observed mainly in younger females, while no difference was observed in the older group.

**Table 2:** Multivariable-adjusted ORs (95 % CI) for female-to-male differences in hypercholesterolemia management by age group (≤50 vs. >50 years) among participants aged ≥15 years in Switzerland, 2002–2022.

**Year**	**2002**	**2007**	**2012**	**2017**	**2022**	***P*-value for interaction (sex×age)**
**Previous screening**						<0.01
Age ≤ 50	0.96 (0.82, 1.13)	0.94 (0.81, 1.10)	1.00 (0.88, 1.13)	1.08 (0.96, 1.22)	1.05 (0.92, 1.20)	
Age > 50	0.88 (0.62, 1.26)	0.87 (0.61, 1.23)	0.57 (0.43, 0.77)	0.85 (0.66, 1.11)	0.56 (0.44, 0.71)	
**Screening last 12 months**						<0.01
Age ≤ 50	1.08 (0.94, 1.25)	1.13 (0.98, 1.31)	1.23 (1.10, 1.38)	1.37 (1.25, 1.51)	1.33 (1.20, 1.47)	
Age > 50	0.91 (0.75, 1.11)	0.98 (0.82, 1.17)	0.86 (0.75, 0.98)	0.81 (0.73, 0.89)	0.93 (0.84, 1.02)	
**Diagnosis**						<0.01
Age ≤ 50	0.52 (0.46, 0.59)	0.52 (0.46, 0.59)	0.62 (0.56, 0.69)	0.64 (0.58, 0.71)	0.62 (0.56, 0.69)	
Age > 50	0.70 (0.62, 0.79)	0.68 (0.60, 0.77)	0.75 (0.67, 0.83)	0.71 (0.64, 0.78)	0.68 (0.62, 0.74)	
**Treatment among diagnosed**						<0.01
Age ≤ 50	0.80 (0.58, 1.09)	0.77 (0.57, 1.04)	0.61 (0.47, 0.81)	0.68 (0.52, 0.88)	0.80 (0.62, 1.05)	
Age > 50	0.79 (0.64, 0.98)	0.64 (0.52, 0.78)	0.64 (0.53, 0.77)	0.79 (0.67, 0.94)	0.75 (0.65, 0.88)	
**Control among treated**						<0.01
Age ≤ 50	2.41 (1.57, 3.70)	2.26 (1.48, 3.44)	1.63 (1.10, 2.41)	2.28 (1.48, 3.53)	1.39 (0.94, 2.04)	
Age > 50	1.03 (0.79, 1.34)	1.02 (0.76, 1.38)	0.89 (0.68, 1.17)	0.83 (0.65, 1.06)	0.88 (0.70, 1.10)	

Statistical analysis using logistic regression, adjusting for nationality, administrative region, educational level, work status, marital status, smoking categories, alcohol consumption categories, physical activity level, and body mass index categories. For the “Age ≤ 50” group, results are expressed as the multivariable-adjusted odds ratio (95 % confidence interval) for younger females (age ≤ 50) relative to younger males (age ≤ 50). For the “Age > 50” group, results are expressed as the multivariable-adjusted odds ratio (95 % confidence interval) for older females (age > 50) relative to older males (age > 50). *P* values for sex×age interaction are from multivariable-adjusted pooled models including a sex×age (≤50 vs. >50 years) cross-product term, with adjustment for survey year and the same covariates as above.

### 3.6 Sensitivity results

IPW analyses yielded similar findings across screening, diagnosis, treatment, and control, except for screening, where females were more likely to have been screened within the last 12 months since 2017, and the lower likelihood of previous screening observed in 2022 was no longer significant (**Supplementary Table 5**).

## 4 Discussion

Using 20 years of nationally representative data, this study indicates age-specific sex disparities across all stages of hypercholesterolemia management in Switzerland, with no evidence of narrowing disparities over time.

### 4.1 Screening

Approximately 90 % of the participants reported having undergone a previous cholesterol screening, higher than rates reported in Geneva (∼70 %) (Marques-Vidal et al., 2023). The differences may stem from variations in regional population demography and differing definitions of screening.

Females were overall less likely than males to report previous screening, consistent with findings from an Australian study, though not with a Dutch study. The Australian study found that females in primary prevention were less likely than males to have CVD risk factor assessment, including cholesterol (Hyun et al., 2017). In contrast, the Dutch study in primary care observed that lipid assessment was more frequently measured in females than males (Kiss et al., 2024). This inconsistency may result from differences between primary and secondary care settings. As highlighted in our study, females aged over 50 were more likely to be under-screened than age-matched males, while no significant differences in screening were observed between sexes aged under 50. This may suggest a potential sex bias among healthcare providers at an age when CVD risk, including cholesterol, should be assessed every five years. Healthcare providers may underestimate CVD risk in females aged over 50 compared to males of the same age, possibly due to fewer visible CVD risk factors. Conversely, the overall higher likelihood of females being screened in the past 12 months was primarily observed in the younger age group starting in 2012. This observation may suggest a relatively higher screening uptake among younger females than among younger males in recent years, potentially driven by increased educational levels, greater health awareness, or enhanced gynecological care. However, it is important to note that both males and females showed a decline in recent screening rates after 2012 compared to before.

Notably, the 2022 survey wave coincided with the post-COVID period, when most restrictions in Switzerland had already been lifted. While a direct impact of the pandemic on cholesterol screening is unlikely, we cannot exclude indirect effects. For example, temporary disruptions of routine care in 2020–2021 may have influenced participants' recall or uptake of screening in 2022. Hence, some of the differences observed in 2022 compared with earlier years could also reflect residual pandemic-related effects.

### 4.2 Diagnosis

Approximately 65 % reported a hypercholesterolemia diagnosis, higher than the prevalence reported in other studies restricted to specific regions of Switzerland, where prevalence ranged from 25 % to 50 % (Marques-Vidal et al., 2023; Rochat et al., 2023). This discrepancy likely reflects heterogeneity in hypercholesterolemia definitions, which remain inconsistent across Swiss healthcare settings.

Females consistently had a lower likelihood of being diagnosed, a finding aligning with previous studies (Marques-Vidal et al., 2023; SAE et al., 2019), showing that hypercholesterolemia prevalence is higher in males (Pinho-Gomes et al., 2020). The lower diagnosis of hypercholesterolemia in females may be attributed to biological factors, such as the protective effects of estrogen, which help maintain lower lipid levels in females (Patel et al., 2024). This hypothesis is supported by our study, which found a more pronounced sex difference in individuals under 50, where females' cholesterol levels are typically lower than males' (Patel et al., 2024). However, if biological factors were the sole explanation, we would expect the sex gap in the older group with less or no difference. This was not the case, suggesting potential sex biases among healthcare providers, who may perceive females as being at lower risk of CVD. This could reflect bias in clinical risk scores used to determine lipid-lowering treatment eligibility and benefit (Delabays et al., 2023).

### 4.3 Treatment

Almost 90 % of the participants diagnosed with hypercholesterolemia were treated, a coverage higher than previously observed in Switzerland (40 %) (Marques-Vidal et al., 2023; Rochat et al., 2023) and in other countries, where it ranged from 25 % to 80 % (SAE et al., 2019; Pinho-Gomes et al., 2020; Nanna et al., 2019).

The lower likelihood of receiving treatment in females diagnosed with hypercholesterolemia, compared to males, is largely consistent with studies across different regions, population demography, settings, outcomes definitions, and periods (Marques-Vidal et al., 2023; Rochat et al., 2023; Kiss et al., 2024; Gheorghe et al., 2020). This lower medication use in females compared to males is unlikely to stem from the belief that young females are protected by estrogen (Patel et al., 2024), as this lower likelihood was present in both young and older females. Other explanations include poorer statin adherence in females due to side effects (Gheorghe et al., 2020), potentially due to their underrepresentation in clinical trials (Farkouh et al., 2020). Older females are more likely to experience polypharmacy (Cebrino and Portero de la Cruz, 2023), which increases the risk of drug interactions with statins, possibly contributing to higher treatment discontinuation in this population. Finally, sex differences in risk perception and health literacy may also contribute to the undertreatment of females, as one study found that females were less likely than males to believe that statins are effective and safe, and more likely to associate statins with diabetes, liver damage, and muscle symptoms (Nanna et al., 2019).

### 4.4 Control

The high rate of achieving cholesterol control among those treated was also observed in the Geneva study (Marques-Vidal et al., 2023), although other studies reported a wide range of control rates, ranging from 20 % to 95 % (Rochat et al., 2023; SAE et al., 2019; Pinho-Gomes et al., 2020).

Females treated for hypercholesterolemia were overall more likely to achieve controlled cholesterol levels than males. Our results revealed discrepancies with previous studies, which generally indicated poorer hypercholesterolemia control among females (Rochat et al., 2023; Kiss et al., 2024; SAE et al., 2019; Pinho-Gomes et al., 2020; Gavina et al., 2023). The discrepancy may be due to variations in the age structure of the study population, with the older median age in the PORTRAIT-DYS study (Gavina et al., 2023), which may result in a higher likelihood of simultaneous use of multiple medications, increasing the risk of side effects in females more than males, leading to poorer treatment adherence (Gheorghe et al., 2020). Our sensitivity analysis revealed that the overall higher likelihood of control among females was driven by the younger age group. In contrast, no significant difference was found between older females and males. This may highlight a biological advantage of estrogen in lipid-lowering efficacy or a generally better lipid profile before menopause. (Patel et al., 2024)

### 4.5 Limitations and strengths

This study has limitations. First, all outcomes were self-reported and lacked clinical validation, which may lead to misclassification and recall bias. This likely explains the higher self-reported diagnosis and control rates compared with regional studies based on laboratory measurements (Marques-Vidal et al., 2023). Second, information on treatment was restricted to current self-reported use and did not capture dose, adherence, or statin potency. Finally, healthcare-seeking behaviors could not be assessed. While the findings may not generalize beyond high-income countries, they provide valuable insights into real-world health perceptions and behavior, informing sex-sensitive prevention strategies and population-level interventions.

This study also offers several strengths. First, it uses five nationally representative Swiss Health Surveys spanning 20 years, providing one of the most comprehensive population-based assessments of hypercholesterolemia management by sex in a high-income country. The large sample size and repeated cross-sectional design enable the identification of temporal trends. Second, the inclusion of a wide range of sociodemographic and lifestyle covariates, along with sensitivity analyses using inverse probability weighting and menopausal age stratification, supports the robustness of observed sex differences.

## 5 Conclusion

In Switzerland, sex differences were observed across all stages of hypercholesterolemia management. Females, particularly older females, were less likely to be diagnosed and treated compared to males. In comparison, younger females were more likely to be screened in the last 12 months from 2012 onwards compared to age-matched males. These findings support sex-sensitive strategies across the care cascade, including public awareness initiatives for females >50 years that highlight the risks of untreated hypercholesterolemia and routine sex-stratified monitoring to track disparities over time.

## Financial disclosure statements

This research did not receive any specific grant from funding agencies in the public, commercial, or not-for-profit sectors.

## CRediT authorship contribution statement

**Shun Yi:** Writing – original draft, Visualization, Investigation, Formal analysis. **Roxane de La Harpe:** Writing – original draft, Visualization, Formal analysis. **Pedro Marques-Vidal:** Writing – review & editing, Data curation, Conceptualization.

## Declaration of generative AI and AI-assisted technologies in the writing process

During the review process of this work, the authors used ChatGPT.4 to improve readability and language. After using this tool/service, the authors reviewed and edited the content as needed and take full responsibility for the content of the publication.

## Declaration of competing interest

The authors declare that they have no known competing financial interests or personal relationships that could have appeared to influence the work reported in this paper.

## Data Availability

The existing datasets used are not publicly available as per the contract, but can be requested by the Swiss Federal Office of Statistics.
