# Supplementary figures and images for: Sex differences in hypercholesterolemia management (2002−2022): evidence from the Swiss National Health Surveys
Source: Prev Med Rep. 2025 Oct 8;59:103266. doi: 10.1016/j.pmedr.2025.103266 (PMC12547935; doi:10.1016/j.pmedr.2025.103266)

## Slide 1
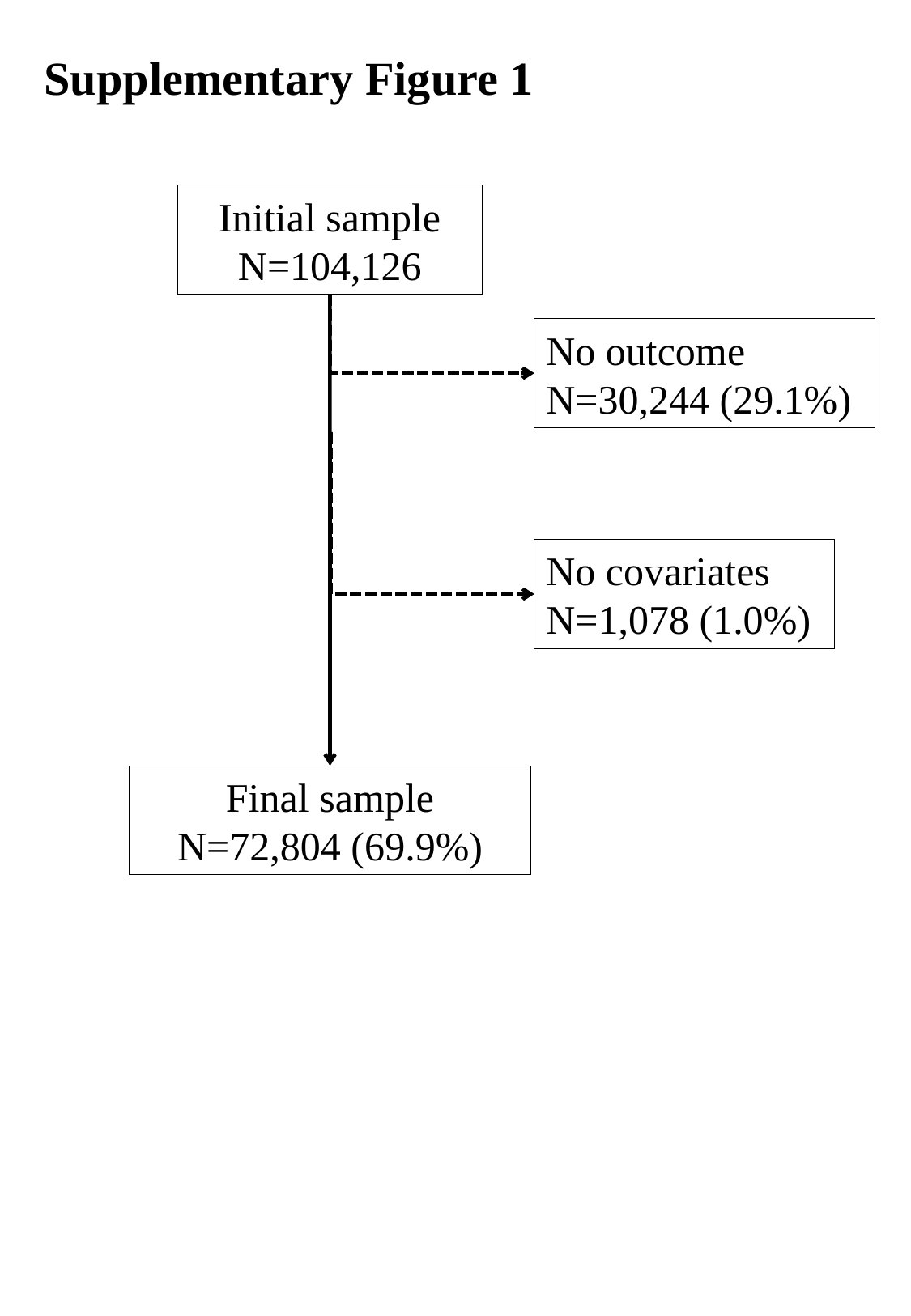

Supplementary Figure 1
Initial sample
N=104,126
No outcome
N=30,244 (29.1%)
No covariates
N=1,078 (1.0%)
Final sample
N=72,804 (69.9%)

Supplement: Supplementary material 1 [file mmc1.pptx]
