# Supplementary material for: Sex differences in hypercholesterolemia management (2002−2022): evidence from the Swiss National Health Surveys
Source: Prev Med Rep. 2025 Oct 8;59:103266. doi: 10.1016/j.pmedr.2025.103266 (PMC12547935; doi:10.1016/j.pmedr.2025.103266)
Supplement: Supplementary material 2 [file mmc2.docx]

# Supplementary figure legends

**Supplementary Figure 1**. Flowchart of sample selection for participants aged ≥15 years in Switzerland, 2002–2022.

Footnote: No outcome refers to participants without valid information on screening. No covariates refers to participants with missing data on one or more covariates (e.g., education, smoking, body mass index).

**Supplementary Tables**

**Supplementary Table 1.** Demographic and lifestyle characteristics of included and excluded participants aged ≥15 years in Switzerland, 2002–2022.

| **Characteristic** | **Included, N (%)** | **Excluded, N (%)** |
| --- | --- | --- |
| **Sample size** | 72,804 | 31,322 |
| **Study year** |  |  |
| 2002 | 11,980 (16.4) | 7,725 (24.7) |
| 2007 | 11,627 (16.0) | 7,133 (22.8) |
| 2012 | 15,422 (21.2) | 6,175 (19.7) |
| 2017 | 16,830 (23.1) | 5.304 (16.9) |
| 2022 | 16,945 (23.3) | 4,985 (15.9) |
| **Region** |  |  |
| Léman | 13,562 (18.6) | 4,664 (14.9) |
| Mittelland | 15,120 (20.8) | 6,636 (21.2) |
| Northwest | 9,471 (13.0) | 4,398 (14.0) |
| Zurich | 7,372 (10.1) | 3,683 (11.7) |
| Eastern | 10,736 (14.8) | 5,096 (16.3) |
| Central | 10,645 (14.6) | 5,186 (16.6) |
| Tessin | 5,898 (8.1) | 1,659 (5.3) |
| **Sex** |  |  |
| Male | 33,600 (46.1) | 14,618 (46.7) |
| Female | 39,204 (53.9) | 16,704 (53.3) |
| **Age group** |  |  |
| 15-24 | 6,913 (9.5) | 4,644 (14.8) |
| 25-44 | 19,488 (26.8) | 11,687 (37.3) |
| 45-64 | 26,872 (36.9) | 9,299 (29.7) |
| 65+ | 19,531 (26.8) | 5,692 (18.2) |
| **Nationality** |  |  |
| Swiss | 60,811 (83.5) | 25,693 (82.1) |
| Other | 11,992 (16.5) | 5,617 (17.9) |
| **Education level** |  |  |
| Primary | 13,344 (18.3) | 6,909 (23.0) |
| Secondary | 38,286 (52.6) | 15,357 (51.1) |
| Tertiary | 21,174 (29.1) | 7,795 (25.9) |
| **Workforce status** |  |  |
| Inactive | 25,367 (34.9) | 9,423 (30.1) |
| Jobless | 1,384 (1.9) | 654 (2.1) |
| Active | 46,034 (63.2) | 21,225 (67.8) |
| **Civil status** |  |  |
| Single | 18,803 (25.8) | 10,634 (34.0) |
| Married | 40,837 (56.1) | 16,093 (51.4) |
| Divorced | 7,775 (10.7) | 2,623 (8.4) |
| Widowed | 5,376 (7.4) | 1,943 (6.2) |
| **Smoking status** |  |  |
| Never | 36,731 (50.4) | 16,573 (53.0) |
| Former | 17,374 (23.9) | 5,918 (18.9) |
| Current | 18,699 (25.7) | 8,790 (28.1) |
| **Alcohol consumption** |  |  |
| No consumption | 12,420 (17.1) | 4,447 (16.4) |
| Low risk | 46,331 (63.6) | 17,557 (64.7) |
| Moderate risk | 10,358 (14.2) | 3,664 (13.5) |
| Average risk | 2,568 (3.5) | 1,028 (3.8) |
| High risk | 1,127 (1.6) | 448 (1.6) |
| **Vigorous physical activity** |  |  |
| None | 26,445 (36.3) | 9,304 (34.6) |
| 1–2 days/week | 25,418 (34.9) | 10,019 (37.3) |
| ≥3 days/week | 20,941 (28.8) | 7,548 (28.1) |
| **BMI category** |  |  |
| Underweight | 2,513 (3.5) | 1,371 (4.6) |
| Normal | 38,968 (53.5) | 18,126 (60.1) |
| Overweight | 23,178 (31.8) | 8,232 (27.3) |
| Obese | 8,145 (11.2) | 2,414 (8.0) |

Results are presented as numbers (percentages) for categorical variables. Between-group comparisons were performed using chi-square tests. BMI, Body Mass Index. Besides cells with a grey background corresponding to non-significant (p≥0.05) results, all comparisons are significant at p<0.05.

**Supplementary Table 2.** Original survey questions on hypercholesterolemia from the Swiss National Health Survey (2002–2022) translated into English.

| **Outcome** | **Question** |
| --- | --- |
| **Screening** | When was the last time your cholesterol level was measured?   - No = “Never” - Yes = range of time ("Within the last 12 months”, “1 to less than 2 years ago”, “2 to less than 3 years ago”, "From 3 to 5 years ago”, “From 5 years ago or more”) |
| **Diagnosis** | - Has a doctor or other medical professional ever told you that your cholesterol levels are too high?   OR   - Presence of cholesterol medication |
| **Treatment** | Please tell me how often you have taken cholesterol medication in the last 7 days:   - Yes = “Every day”, “Several times” or “About 1 time in the last 7 days” - No = “Never” |
| **Control** | - Are your current cholesterol levels normal (= controlled) or too high (= uncontrolled)? |

**Supplementary Table 3.** Sex-specific distribution of hypercholesterolemia management among participants aged ≥15 years in Switzerland, 2002–2022.

|  | **2002** |  | **2007** |  | **2012** |  |
| --- | --- | --- | --- | --- | --- | --- |
| **Characteristic** | **Male, N (%)** | **Female, N (%)** | **Male, N (%)** | **Female, N (%)** | **Male, N (%)** | **Female, N (%)** |
| **Screening** |  |  |  |  |  |  |
| Previous screening | 4,808 (91.0) | 6,065 (90.6) | 4,631 (90.3) | 5,796 (89.2) | 6,478 (88.4) | 7,045 (87.1) |
| Screening last 12 months | 2,946 (74.9) | 3,778 (75.2) | 2,823 (72.7) | 3,692 (73.3) | 3,808 (62.6) | 4,229 (62.8) |
| **Diagnosis** | 3,784 (71.6) | 4,003 (59.8) | 3,515 (68.6) | 3,666 (56.4) | 5,133 (70.0) | 4,911 (60.7) |
| **Treatment** |  |  |  |  |  |  |
| Among diagnosed participants | 3,445 (91.0) | 3,540 (88.4) | 3,168 (90.1) | 3,174 (86.6) | 4,713 (91.8) | 4,373 (89.1) |
| **Control** |  |  |  |  |  |  |
| Among treated participants | 3,171 (92.1) | 3,314 (93.6) | 2,948 (93.1) | 3,001 (94.6) | 4,458 (94.6) | 4,190 (95.8) |
|  | **2017** |  | **2022** |  |  |  |
| **Screening** |  |  |  |  |  |  |
| Previous screening | 7,194 (89.6) | 7,805 (88.7) | 7,146 (91.3) | 8,207 (90.0) |  |  |
| Screening last 12 months | 4,579 (57.0) | 5,017 (57.0) | 4,632 (59.2) | 5,518 (60.5) |  |  |
| **Diagnosis** | 5,586 (69.5) | 5,369 (61.0) | 5,329 (68.1) | 5,289 (58.0) |  |  |
| **Treatment** |  |  |  |  |  |  |
| Among diagnosed participants | 5,086 (91.1) | 4,788 (89.2) | 4,737 (88.9) | 4.588 (86.8) |  |  |
| **Control** |  |  |  |  |  |  |
| Among treated participants | 4,813 (94.6) | 4,580 (95.7) | 4,443 (93.8) | 4,339 (94.6) |  |  |

Results are presented as numbers (percentages) for categorical variables. Between-group comparisons were performed using chi-square tests. Besides cells with a grey background corresponding to non-significant (p≥0.05) results, all comparisons are significant at p<0.05.

**Supplementary Table 4.** Multivariable-adjusted ORs (95% CI) for female-to-male differences in hypercholesterolemia management among participants aged ≥15 years in Switzerland, 2002–2022.

| **Year** | **2002** | **2007** | **2012** | **2017** | **2022** |
| --- | --- | --- | --- | --- | --- |
| **Screening** |  |  |  |  |  |
| Previous screening | 0.95 (0.82, 1.09) | 0.91 (0.79, 1.04) | 0.91 (0.81, 1.02) | 1.02 (0.92, 1.14) | 0.89 (0.79, 1.00) |
| Screening last 12 months | 1.01 (0.90, 1.13) | 1.03 (0.93, 1.15) | 1.05 (0.96, 1.14) | 1.06 (0.99, 1.14) | 1.08 (1.01, 1.16) |
| **Diagnosis** | 0.61 (0.55, 0.66) | 0.61 (0.56, 0.66) | 0.69 (0.64, 0.74) | 0.69 (0.64, 0.74) | 0.66 (0.61, 0.70) |
| **Treatment** |  |  |  |  |  |
| Among diagnosed participants | 0.80 (0.67, 0.96) | 0.68 (0.58, 0.81) | 0.64 (0.55, 0.75) | 0.76 (0.66, 0.87) | 0.77 (0.68, 0.88) |
| **Control** |  |  |  |  |  |
| Among treated participants | 1.31 (1.06, 1.63) | 1.37 (1.08, 1.73) | 1.10 (0.88, 1.37) | 1.10 (0.89, 1.35) | 0.99 (0.82, 1.20) |

Results are expressed as the multivariable-adjusted OR and (95% CI) for females relative to males. Statistical analysis using logistic regression, adjusting for age categories, nationality, administrative region, educational level, work status, marital status, smoking categories, alcohol consumption categories, physical activity level, and body mass index categories.

**Supplementary Table 5**: Multivariable-adjusted ORs (95% CI) for female-to-male differences in hypercholesterolemia management using inverse probability weighting among participants aged ≥15 years in Switzerland, 2002–2022.

| **Year** | **2002** | **p-value** | **2007** | **p-value** | **2012** | **p-value** |
| --- | --- | --- | --- | --- | --- | --- |
| **Screening** |  |  |  |  |  |  |
| Previous screening | 0.95 (0.82, 1.10) | 0.518 | 0.90 (0.78, 1.03) | 0.131 | 0.92 (0.82, 1.03) | 0.131 |
| Screening last 12 months | 1.01 (0.90, 1.13) | 0.852 | 1.03 (0.92, 1.15) | 0.593 | 1.06 (0.97, 1.15) | 0.184 |
| **Diagnosis** | 0.60 (0.54, 0.65) | <0.001 | 0.60 (0.55, 0.66) | <0.001 | 0.68 (0.63, 0.74) | <0.001 |
| **Treatment** |  |  |  |  |  |  |
| Among diagnosed participants | 0.80 (0.67, 0.95) | 0.012 | 0.69 (0.58, 0.82) | <0.001 | 0.64 (0.55, 0.75) | <0.001 |
| **Control** |  |  |  |  |  |  |
| Among treated participants | 1.37 (1.10, 1.71) | 0.005 | 1.38 (1.10, 1.74) | 0.006 | 1.11 (0.89, 1.39) | 0.352 |
| **Year** | **2017** | **p-value** | **2022** | **p-value** |  |  |
| **Screening** |  |  |  |  |  |  |
| Previous screening | 1.02 (0.92, 1.14) | 0.673 | 0.89 (0.80, 1.01) | 0.061 |  |  |
| Screening last 12 months | 1.08 (1.00, 1.15) | 0.042 | 1.10 (1.02, 1.18) | 0.009 |  |  |
| **Diagnosis** | 0.68 (0.64, 0.73) | <0.001 | 0.66 (0.61, 0.70) | <0.001 |  |  |
| **Treatment** |  |  |  |  |  |  |
| Among diagnosed participants | 0.75 (0.65, 0.86) | <0.001 | 0.78 (0.68, 0.89) | <0.001 |  |  |
| **Control** |  |  |  |  |  |  |
| Among treated participants | 1.11 (0.90, 1.36) | 0.332 | 1.00 (0.83, 1.21) | 0.990 |  |  |

Results are expressed as the multivariable-adjusted odds ratio and (95% confidence interval) for females relative to males. Statistical analysis using logistic regression, adjusting for age categories, nationality, administrative region, educational level, work status, marital status, smoking categories, alcohol consumption categories, physical activity level, and body mass index categories.
